# Supplementary material for: Analysis of chi angle distributions in free amino acids via multiplet fitting of proton scalar couplings
Source: Magn Reson (Gott). 2024 Aug 19;5(2):103–20. doi: 10.5194/mr-5-103-2024 (PMC11570886; doi:10.5194/mr-5-103-2024)
Supplement: The supplement related to this article is available online at: https://doi.org/10.5194/mr-5-103-2024-supplement. [file mr-5-103-2024-supplement.zip › Syed2024/data/fit1d_fitnmr_output.tar.gz!/fit1d/lys/500/bmse000043_simulation_1_nmredata/1H/sparse_1d.pdf]

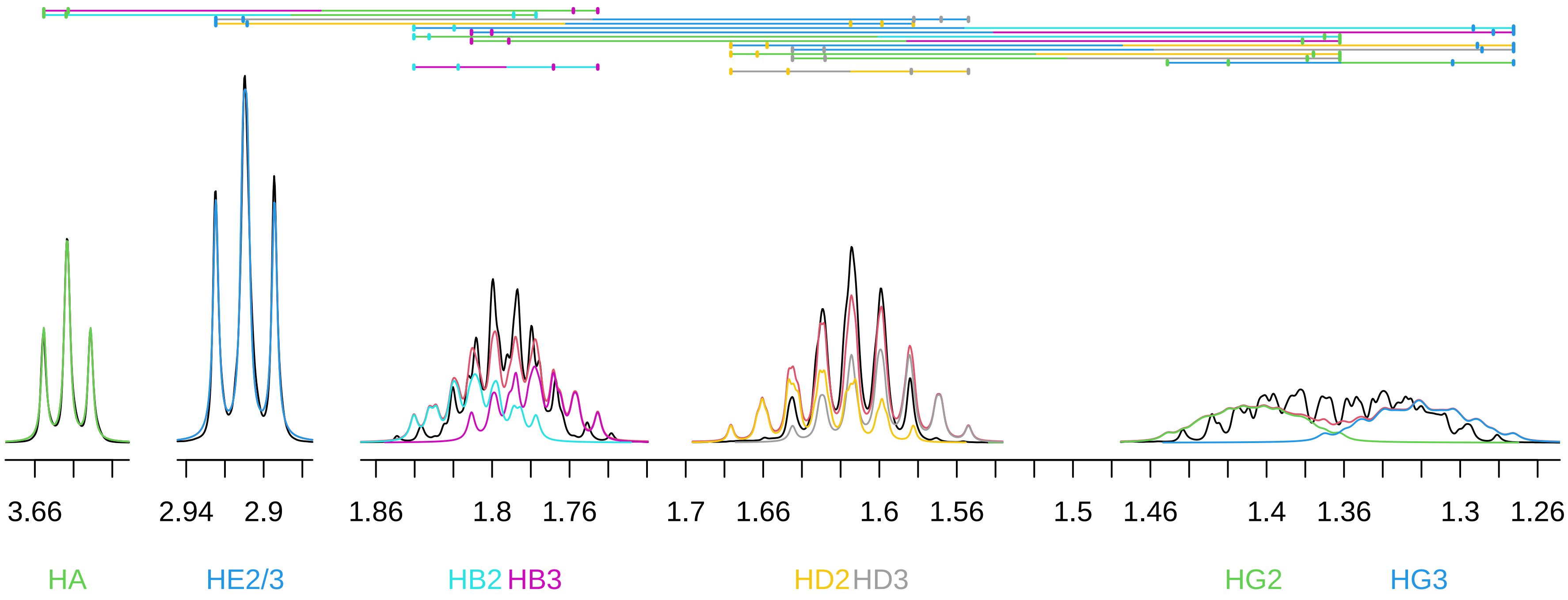

3.66
2.94
2.9
1.86
1.8
1.76
1.7
1.66
1.6
1.56
1.5
1.46
1.4
1.36
1.3
1.26
HA
HE2/3
HB2 HB3
HD2 HD3
HG2
HG3
